# Writing letters to patients as an educational tool for medical students

**DOI:** 10.1186/1472-6920-13-114

**Published:** 2013-08-23

**Authors:** Nataša Mrduljaš Đujić, Edi Žitnik, Ljubica Pavelin, Dubravka Bačić, Mia Boljat, Davorka Vrdoljak, Ivančica Pavličević, Ana Radica Dvornik, Ana Marušić, Matko Marušić

**Affiliations:** 1 Department of Family Medicine, University of Split School of Medicine, Šoltanska 2, Split 21000, Croatia; 2 ‘Mi’ Association and ‘Kajo Dadic’ Foundation Split, Split, Croatia; 3 Croatian Institute for Pension Insurance, Split, Croatia; 4 Department of Research in Biomedicine and Health, University of Split School of Medicine, Split, Croatia

**Keywords:** Doctor-patient communication, Student-patient relationship, Letter to patient, Family medicine

## Abstract

**Background:**

Despite rapid growth and development of medical technology, personal relationship between the patient and physician remains the basis of high quality treatment. The aim of our study was to develop, implement and evaluate patient therapeutic letters written by students as a tool in teaching family medicine.

**Methods:**

The study included all 6^th^ year students attending their rounds in family medicine, structured into two 10-day cycles, one in urban offices and one in offices on the Adriatic islands (rural). After receiving detailed instructions, students wrote letters to two patients after a consultation in the office. The letters were audited by patients and 3 family medicine experts who used a grading instrument (scale 0 – poor, 1 – medium, 2 – good) for 1) adequacy and clarity of description of patients’ disease/state, 2) knowledge, 3) adequacy of recommendations, 4) courtesy and respect and 5) language and style. Patients and experts were also asked to underline phrases they thought would be difficult to understand; the underlined text was subjected to content analysis.

**Results:**

Both the patients and the experts gave high scores for the value and quality of the letters in terms of the description of the problem, adequacy of recommendations given, and courtesy and respect (mean (±standard deviation) 5.65 ± 0.79 for patients vs. 4.87 ± 0.79 for experts out of maximum score of 6). Family medicine experts were stricter than patients in their evaluation of the content of the letters (adequacy and clarity of disease description (P < 0.001) and adequacy of recommendations (P < 0.001). Both the patients and the experts seemed to like longer letters as the length of the letter showed significant positive correlation with the quality summary score (correlation r = 0.492 vs. r = 0.338, respectively, P < 0.010). Overlapping of the text underlined as difficult to understand by patients and experts was found in 10 (11.6%) out of 86 letters. The highest overlap (20 terms) was found for the category “Technical terms unclear to a lay reader”.

**Conclusions:**

Writing of a letter to their first patients may be a useful tool for students to personally experience the practice of medicine and establish better partnership with patients in health care.

## Background

*“The hardest conviction to get into the mind of a beginner is that the education upon which he is engaged is not … a medical course, but a life course, for which the work of a few years under teachers is but a preparation”* Sir William Osler (1849–1919), from “The Student of Medicine”

Despite rapid growth and development of medical technology, personal relationship between the patient and physician remains the basis of high quality treatment [1]. This is particularly important in family medicine, which has a unique role of providing continuity and coordination of, as well as trust in health care for the patients [2,3]. Poor communication may result in delayed provision of adequate diagnosis or treatment and sometimes lead to malpractice allegations [4]. In recent years, teaching communication skills as a part of professional development has become integrated in many medical curricula [5,6], using the development of a relationship with a patient as a way of learning in health care settings, encouraging students to build their confidence and self-esteem and learn how to apply their knowledge in personalized and individualized care.

Most educational interventions in communication skills, particularly in family medicine [7], focus on students’ consultations with patients. There is less evidence for the use of written communication with patients as a way to train students for building therapeutic relationship with them [8]. Written correspondence in medicine is usually reserved for communication between physicians [9]. The educational value of written communication has been explored in nursing, where therapeutic letters proved to be a powerful strategy for student learning [10,11].

There are only a few reports of using letter writing as an educational tool for health professionals. A long-term project of letters exchanged between medical students, community teenagers and terminally ill patients was appreciated as a good way of establishing bidirectional communication [8]. This communication served as an educational and mentoring experience for patients, as well as important reflective exercise in accepting and living with their disease: “Writing to you has allowed me to reconnect with parts of myself that I had forgotten about or thought I had lost, so thank you for that”. [8]. For students, the writing exercise was a way for personal maturation and learning how to talk about sensitive topics under difficult circumstances. In another project of letters written by nurses to the families of patients, such interventions were shown to have important effect on communication skills of the students and recognition of patient and family strengths in managing the disease [11].

Letters are thus reminiscent of the teaching during Renaissance, when “Observations” – i.e. description of cases were documented in the 16th century as a primary way of communication and learning medicine [12].

The aim of our study was to develop, implement and evaluate the use of patient therapeutic letters written by medical students during their rotations in family medicine (FM) offices, as a complementation to already established training in communication skills during FM consultations. The letters were audited by patients and FM experts, and their content analyzed to identify the phrases or themes that may generate communication problems for the patients. Our study extends the research into letters as a form of communication with patients into two new avenues: 1) as a part of communication within the set-up of a consultation in family medicine practice, in contrast to the relay of specialty information to the patient by the family physician, and 2) as an education tool for medical students in family medicine course. We also explored different aspects of the written communication, including both the professional content and the communication style.

## Methods

### Setting

The study was conducted in FM practices during the FM rounds at the 6th year of the medical curriculum at the University of Split School of Medicine, in 2011. FM rounds in the School’s curriculum are structured into 2 cycles of 10 days each, one in city FM offices and one in FM offices on the Adriatic islands (rural FM offices).

### Subjects

The study included all 6th year students attending their rounds in FM in 2011 (n = 54). The instrument we developed for grading students’ letter to patients was also tested on the sample of 64 students attending 4^th^ year of the curriculum in 2011 (all students attending Internal Medicine course). Letter writing assignment was compulsory and was a requirement to pass the course. Both groups of students received the same instructions and underwent the same assessment.

The study was approved by the Ethics Committee of the University of Split School of Medicine.

### Consultation

During their FM practice, the students conducted consultations with visiting patients. Under supervision, they took medical history and performed a complete physical examination, explained to the patients the findings and provided therapy and/or life-style recommendations.

Upon completion of the consultation, the supervisors asked the patients to assess student’s competence to deal with the patients’ health problem by filling in a standardized questionnaire, Patient Enablement Instrument (PEI) [13]. As an outcome measure, PEI demonstrates the extent to which patients understand their medical problems and are able to deal with them. It contains six questions (able to cope with life, able to understand his/her illness, able to cope with his/her disease, able to keep himself/herself healthy, strong confident in his/her health and able to help himself/herself), with a score range of 1 to 3 points (much better, better, equal or worse) and maximum of 18 points. The supervisor also recorded the length of the consultation.

### Students’ letters to patients

During their FM rotation, students chose two patients from their consultations and wrote letters to them after the consultation, following detailed written instructions on how to structure the letters: 1) basic information about the patient: age, gender, employment status and diagnosis; 2) symptoms and reasons for the visit to the FM office 3) description of the findings from the consultation and patient’s general health condition; 4) student’s view of the patient’s actual problem and facts about the disease or symptoms and 5) recommendations to the patient about their treatment and lifestyle related to the disease [14]. The choice of patients was made jointly by a student and his or her tutor. The tutor was provided with the following selection criteria: 1) inclusion criteria – any patient with a chronic disease, capable of verbal and written communication, regularly visiting FM office on his or her own; 2) exclusion criteria – patients who could not read or write, those mentally incapacitated, suffering from a psychiatric disease with decreased mental competence and/or official custodian, and with disturbance of consciousness any kind.

The preferred format of the letter was 2 pages (spacing 1.5, font Times New Roman 12). The letter had to be written up to two weeks after consultations, and had to be submitted to the supervisor, who then delivered it to the patient. The patients were asked to read the letter at home, evaluate it and underline words, phrases or sentences they found difficult to understand. They returned the letter to the supervisor within a week.

Student’s letters were independently evaluated by 3 FM specialists from the Department of Family Medicine, using an evaluation instrument similar to that filled in by the patients. The evaluators were blinded to the identity of the student, as the letters were coded by one of the authors (NMD). Students were informed that their letters will be scored by experts, and had to possibility to see the letters after evaluation, as a part of the course evaluation.

### Development of instruments for the evaluation of students’ letters

We developed a grading instrument for the evaluation of students’ letters. The scoring included 5 categories: 1) *adequacy and clarity of description of* patients’ disease/state, 2) *knowledge*, 3) *adequacy of recommendations*, 4) *courtesy and respect* and 5) *language and style*. Each category was graded on a scale from 0 to 2 points (poor, medium and well; possible score range 0–10 points for 5 categories). To test the face validity of the instrument we asked 10 FM experts to pick up 5 out of 10 proposed evaluation items, according to the estimated importance of these categories for assessing the quality of patient letters. They were consistent in their choices so the final instrument contained the above-described 5 categories.

Three categories from the same instrument were used for patients’ grading of the letters: 1) adequacy and clarity of description of their disease/state, 2) adequacy of recommendations, and 3) courtesy and respect (scale range 0–2, possible score range 0–6).

We also validated the scoring instrument on 2010/11 generation of the 4^th^ medical students, using the same format of the questionnaire but extending the scoring range from 0–2 to 1–5 (unsatisfactory, satisfactory, good, very good, excellent).

### Content analysis

Patients were informed by their family physician (student’s tutor) about their role in evaluating students’ letters at the time of entering the study (signing informed consent). They were asked to underline the parts of the letters they did not understand. The experts also underlined phrases in the letters according to the following criteria: 1) *unsuitable phrases* (examples. “I don’t want to scare you by my letter but remind you…”, “it would be good to tidy your home and get rid of cigarette smell”), 2) *technical terms unclear to the lay reader* (examples: “endoscopic sphincterectomy …”, “cardiomyopathia ischaemica compensata …”, “3) *insufficient or vague recommendation* (examples: “exercise which you adapted to your physical fitness …”, “don’t oversalt your dishes …”), 4) *grammatical and spelling errors*, 5) *ignorance and incorrect statements* (examples: “in this way we treat both the viral and bacterial causes…”, “it would be best to open a long sick-leave …”), 6) *overt servility to the patient* (examples: “I granted you your wish and wrote a referral slip for the hospital …”, “As a sign of my support, I give you this device for measuring blood glucose levels…”). The phrases were entered into text-analysis software.

### Statistics

Statistical analysis was performed on a sample of 108 letters to patients of 54 students. Missing variables were excluded for analysis (range of valid data for each variable was 80–108). Scoring instrument was also validated on a sample of 255 letters written by 64 students from 2010/11 generation of the 4^th^ study year. Data were presented as a mean score ± standard deviation (SD).

Student *t*-test was used to test the difference between the variables, and Pearson correlation coefficient for correlation between variables. The agreement between experts was tested by Kendall’s coefficient of concordance W, and the differences between them by Friedman test.

Chi-square test was used to analyze associations of the evaluations with regard to patients’ gender, occupation, level of education, and place of the doctor’s office (rural vs. urban).

Discriminant function analysis [15] was used to determine which variables discriminated between the patients from rural and urban FM offices.

## Results

The final sample included 108 letters to patients from all 6th year students (n = 54) attending their FM rounds in 2011. Letter scoring instrument was also validated on the sample of 255 letters from 64 students of the 4^th^ study year in 2011/2012 academic year (all students who attended the Internal Medicine course). The students were predominately women (41 (76%) of 6th year students and 39 (61%) of 4^th^ year students). The mean age was 23.9 ± 1.1 for the 6^th^ year students and 22.4 ± 1.7 for the 4^th^ year students.

### Experts’ and patients’ evaluation of students’ letters to patients

We first evaluated the scoring instrument for expert physicians by analyzing the concordance of the expert’s evaluation of students’ letters (Table 1). Although their scores differed for all scoring categories (one expert scored consistently lower than the other two; Table 1), the experts were concordant in 3 out of 5 scoring categories: “Knowledge”, “Adequacy of recommendations” and “Courtesy and respect”, as well as their overall evaluation. There were no zero scores. Due to the high level of concordance in their evaluations and statistically significant correlation between experts’ evaluations (Pearson’s correlation coefficient (r) range 0.227 – 0.588, P ≤ 0.004 for all coefficients), we used the evaluations of all three in further analysis of the scoring assessment. When we retested the instrument on another cohort of students, using the scoring range from 1 (“worst”) to 5 (“best”) points (similar to grade points in Croatian medical curriculum), there was still significant concordance among the same three physician experts, now in all scoring categories, and the experts’ scores also differed for all categories, except for precision and language score (Table 2).

**Table 1:** **Concordance of experts in their assessment scores of letters to patients written by 6**^**th **^**year students attending family medicine rotation in 2011**

**Evaluation category**	Score (mean ± SD)*			**Concordance†**	**Difference‡**
	**Expert 1**	**Expert 2**	**Expert 3**	**W (P)**	***χ***^**2 **^**(P)**
Adequacy and clarity of description of disease/state	1.43 ± 0.57	1.36 ± 0.48	1.75 ± 0.49	0.372 (0.197)	32.525 (<0.001)
Knowledge	1.47 ± 0.55	1.28 ± 0.53	1.74 ± 0.48	0.484 (0.002)	45.529 (<0.001)
Adequacy of recommendations	1.43 ± 0.58	1.50 ± 0.52	1.80 ± 0.40	0.469 (0.004)	34.740 (<0.001)
Courtesy and respect	1.91 ± 0.32	1.62 ± 0.51	1.86 ± 0.42	0.413 (0.047)	31.442 (<0.001)
Language and style	1.78 ± 0.44	1.58 ± 0.53	1.53 ± 0.57	0.357 (0.288)	14.588 (<0.001)
Total score	8.02 ± 1.37	7.34 ± 1.46	8.69 ± 1.66	0.519 (0.001)	64.153 (<0.001)

*The scale ranged from 0 to 2 points (maximum 10 points for the five evaluated categories). *SD* standard deviation.

^†^Kendall’s W concordance test.

^‡^Friedman’s test.

**Table 2:** **Concordance of experts in their assessment scores of letters to patients written by 4**^**th **^**year students in 2011**

**Evaluation category**	Score (mean ± SD)* (N = 363)			**Concordance†**	**Difference‡**
	**Expert 1**	**Expert 2**	**Expert 3**	**W (P)**	***χ***^**2 **^**(P)**
Adequacy and clarity of description of disease/state	3.87 ± 1.04	2.80 ± 0.71	4.06 ± 0.91	0.490 (<0.001)	19.070 (0.006)
Knowledge	3.87 ± 1.05	2.95 ± 0.76	4.45 ± 0.88	0.547 (<0.001)	25.009 (0.003)
Adequacy of recommendations	3.48 ± 1.18	2.74 ± 0.76	2.75 ± 1.26	0.576 (<0.001)	24.662 (0.017)
Courtesy and respect	4.15 ± 0.95	3.87 ± 0.48	4.75 ± 0.52	0.456 (<0.001)	7.286 (0.023)
Language and style	3.87 ± 1.07	3.69 ± 0.57	4.48 ± 0.68	0.473 (<0.001)	2.823 (0.361)
Total score	19.25 ± 4.85	16.06 ± 2.51	20.50 ± 3.01	0.582(<0.001)	263.064 (0.020)

*The scale ranged from 1 to 5 points (maximum 25 points for the five evaluated categories). *SD* standard deviation.

^†^Kendall’s W concordance test.

^‡^Friedman’s test.

Patients scored students’ letter significantly higher than experts in all three categories common to both scoring instruments (Table 3). Patients’ evaluations did not differ with respect to patients’ sex, level of education, occupation and urban or rural placement of the doctors’ offices (P > 0.05 for all comparisons, Chi-square (*χ*^2^) test).

**Table 3:** Comparison of evaluations of students’ letters to patients by three expert physicians and patients for three identical categories in the scoring instrument

**Evaluated categories**	Score (mean ± SD)*		**t (P)†**
	**Experts**	**Patients**	
Adequacy and clarity of description of disease/state	1.51 ± 0.34	1.85 ± 0.39	5.86 (<0.001)
Adequacy of recommendations	1.55 ± 0.34	1.87 ± 0.37	6.01 (<0.001)
Courtesy and respect	1.80 ± 0.29	1.93 ± 0.25	3.15 (0.002)
Total score	4.87 ± 0.79	5.65 ± 0.79	6.75 (<0.001)

*The scale ranged from 0 to 2 points for individual categories and 0 to 6 point for the total score. SD standard deviation.

†Student *t*-test.

### Evaluation of students’ consultations with the patients

The consultations of students with patients in the rural offices lasted longer that in the urban offices (mean ± SD: 29.3 ± 2.0 min in rural and 22.9 ± 3.0 min in urban offices; t = 2.46, P = 0.016). There were no differences with respect to the age or sex of the patients (data not shown). There was a significantly positive correlation of the length of consultation (total average 25.9 ± 12.1 min) and PEI score (total average 12.7 ± 2.6 points out of maximum 18 points, r = 0.321, P < 0.01). Significantly higher enablement was found among employed patients in comparison to retired patients (13. 7 ± 2.3 vs. 11.9 ± 2.5; t = 2.785, P = 0.007).

Stepwise discriminant analysis of the patients’ characteristics (age, sex, employment status and education level) and their scoring of the letters, length of consultation and PEI score, showed that older patients from island FM offices had longer consultation, whereas longer consultation in the city offices were more common for middle-aged patients (Wilk’s λ = 0.797, P = 0.008, with 61.5% correctly classified patients of each group). Two variables in combination (consultation length and age of patients) were best discriminators of patients from rural (n = 54) and urban (n = 54) FM offices.

### Content analysis

The average number of words per letter in the sample of 108 letters was 731.1 ± 384.1 (mean ± SD). One expert did not underline any words in the letters, so the content analysis was performed on the data available from 2 experts and the patients.

There was a statistically significant correlation between the length of letters and mean evaluation score of either the 2 experts (r = 0.492) or patients (r = 0.338, p < 0.01).

Patients and experts underlined words or phrases in 86 letters; on average, the percentage of underlined text per letter was 7.1 ± 7.3 (mean ± SD).

Table 4 shows the coding of the underlined phrases into 6 groups of phrases. Experts underlined three times more terms than patients. They also significantly differed in the categories of underlined phrases (χ^2^_5_ = 37.471, P < 0.001): whereas patients mostly underlined words they thought too technical (64.5% of the phrases) and did not note grammatical errors at all, the experts were less worried about technical language (38.1% of the phrases) or vagueness of the recommendations (20.4% vs. 11.2% phrases underlined by patients) but noted more grammatical errors and incorrect statements.

**Table 4:** Content analysis of coded references in students’ letter underlined by experts and patients

**Coding category**	**No. underlined terms**			
	**Patient**	**Expert 1**	**Expert 2**	**Expert 1 + 2 (de-duplicated)**
Unsuitable phrases	14 (13.1%)	46	42	81 (22.4%)
Technical terms unclear to a lay reader	69 (64.5%)	99	55	138 (38.1%)
Insufficient or vague recommendations	11 (10.3%)	5	14	18 (5.0%)
Grammatical and spelling errors	0 (0.0%)	32	14	42 (11.6%)
Lack of knowledge or incorrect statements	12 (11.2%)	45	35	74 (20.4%)
Overt flattery to the patient	1 (0.9%)	5	5	9 (2.5%)
Total	107 (100%)	232	165	362 (100%)

Phrases underlined in the text by patients and experts did not overlap to a great extent. Overlapping was found in 10 (11.6%) out of 86 letters. There was no overlap in the categories “Insufficient or vague recommendations”, “Grammatical and spelling errors” and “Overt flattery to patients”, whereas the highest overlap (20 terms) was found for the category “Technical terms unclear to a lay reader”. Small overlap existed also for 4 phrases in the category “Lack of knowledge or incorrect statements” and 3 phrases in the category “Unsuitable phrases”.

## Discussion

This study explored the reliability and quality of letter-writing by medical students to the patients they saw during consultations as a teaching tool in family medicine. Both the patients and the experts gave high scores for the value and quality of the letters in terms of the description of the problem, adequacy of recommendations given, and courtesy and respect.

The patients, regardless of their age, gender, education level or place of living, gave higher scores than family medicine experts for all criteria – description of disease, recommendation and courtesy, most probably because that they valued the novel way of learning about their disease and recommendations for therapy and lifestyle change.

Family medicine experts were much stricter than patients in their evaluation of the content of the letters (adequacy and clarity of disease description and adequacy of recommendations), indicating that they focused on the transfer of medical facts. On the other hand, the highest scores and the smallest difference between the patients and experts were for courtesy and politeness, demonstrating that this aspect of patient-physician communication is the basic premise for interpersonal relationships in a family medicine office [16].

Both the patients and the experts seemed to like longer letters, as the length of the letter showed significant positive correlation with the given quality summary score.

The results of our study are limited by its cross-sectional design, lack of a control group, and absence of objective outcome, but the rating of the letters by experts and their agreement on the scores was consistent across two different student cohorts. Furthermore, the selection of the patients, which was made by the students and their tutors according to very general criteria provided by the researchers to the tutor, could have influenced the results because students and tutor may have chosen the patients they felt most confident to describe in a letter. As students had different tutors in urban and rural FM offices and we did not find differences in the quality of letters between these two sites, it is unlikely that students systematically chose more “convenient” patients for their letters. As the letters were a part of the course evaluation, it is more likely that they followed their tutors’ instructions and found most adequate patients for the task.

Within these limitations, our study indicates that the exercise of letter writing is a reliable education tool for family medicine course to develop competencies of students for communication with patients. Based on our first experience of writing letters to patients as a mandatory part of the family medicine course, we will continue to evaluate this educational intervention using more stringent methodological designs, and explore further how written communication would best supplement other learning tools in family medicine training [14].

The results of our study could also be interpreted as evidence for paternalistic views of family medicine practitioners, who prefer providing instruction and clinical competencies to communication with patients. The dominance of such paternalistic approach takes away the patient from the central role and from advantages of the “partnership in care” and “joint partnership” for increasing health care quality [17]. The patient is a passive receiver of instructions, advice and information, with little respect for his or her feelings, opinions and priorities. Such traditional approach to family medicine should be replaced by the use of best available evidence coupled with the patient’s personal choice and values [17]. However, such change can be best accomplished by radical reform of graduate medical education, with emphasis on empathy and communication skills [18]. The reform can then bring about the development of different educational interventions for practical competencies, such as communication labs which help students to understand their future role as physicians and their relationship with patients, to develop self-confidence and prepare them for the first contact with patients [19,20]. The curricular reforms are directed to increasing practical work, problem solving, early contact with patients and greater bonding of students with their mentors [20,21]. The relationship between the student and the teacher should mirror patient-physician relationship: it should have the characteristics of mutual respect and build the process of shared decision-making, with the aim of promoting cooperation rather than competition [22].

Direct evidence for the value of written communication with the patients comes from the study of Roberts and Partridge (2006) [23], which compared the satisfaction of patients and family medicine practitioners in receiving letters from outpatient consultants written specifically for the physician or for the patient. While patients appreciated both types of letters, they found significantly more terms they did not understand in the letters written to physicians than to patients. The letters written specifically for patients were also significantly shorter and easier to read.

In contrast to the study of Roberts and Partridge [23], where patients underlined more terms they could not understand, family medicine experts in our study underlined three times more problematic phrases than patients. While patients were mostly concerned with phrases they thought were not clear for a lay reader, the experts were equally concerned with correct professional terminology and correctness of information as they were with the adaptation of the style for the lay reader. Together with the value ratings of the letters, our results show that patients appreciated the letters as a benefit of their visit to the family medicine office. This was a general experience, as it was not influenced by the characteristics of the patients, including age, gender, education and employment. The type of family practice (urban vs. rural) also did not influence the evaluation of letters, although the consultations in rural offices were significantly longer than in urban offices and this was also significantly associated with higher patient enablement. Our qualitative analysis of patients’ reaction to letters [7] showed that some patients were initially anxious about getting a letter from their consultation in a family medicine office, as they are used to often getting bad news from specialists’ consultations of discharge letters from hospitals. However, after the experience, their responses were mostly positive and optimistic, not only from the point of view of information received (“I understood everything in the letter and now I know about my disease better than before.”) but a personal satisfaction and hope (“I periodically go back to read the letter again, and it helps me.”, “I found the letter very comforting and reassuring.”) [24]. We are currently working on a separated qualitative analysis of students’ evaluation of the letter-writing exercise to explore their satisfaction with the learning outcomes, interactions with the faculty and professionalism.

## Conclusions

Taken together, our results suggest that writing letters to patients by medical students is beneficial for both the students and the patients. For a patient, the letters open a new line of communication outside of routine verbal consultation in the office, which is emotionally coloured and difficult to remember [25,26]. For older patients especially, a written letter may provide better instruction and be read again in peace when the patient is less anxious and more composed. The letter may also help the patient to communicate information to other members of the family [26]. For students, writing letters helps build students communication and empathy skills. The letters enable students to first reflect on their own experience and then share it with the patients, where they are often in the role of mentors for students [27], particularly during the first real contact of students with the patients in community setting, i.e. family medicine office.

Our current research is focused on a longitudinal study of students who write letters to patients, to explore the effect on the development of empathy in students [28], as well as to assess the objective outcomes and subjective experience in letter writing and feedback from the patients. We hope that the ongoing longitudinal study will help us understand whether writing of a letter to their first “real” patients would help students to personally experience the practice of medicine, and provide an experience that students may remember and use in practice longer than many “standard” learning outcomes.

## Ethical approval

The study was approved by the Ethics Committee of the University of Split School of Medicine.

## Competing interests

The authors declare that they have no competing interests.

## Authors’ contributions

AM and MM contributed to the conception and design of the study, analysis and interpretation of data and drafting of the manuscript revising it critically for important intellectual content. NMD contributed to the conception and design of the study, collection, analysis and interpretation of data, and drafting of the manuscript revising it critically for important intellectual content. EŽ contributed to analysis and interpretation of data and the revision of the manuscript. LP, DB, MB and DV contributed to collection, analysis and interpretation of data, and critical revision of the manuscript. IP contributed to collection and analysis of data, and critical revision of the manuscript. All authors approved the submitted version of the manuscript.

## Authors’ information

NMĐ, Office of FM Postira, Brač

LP, Office of FM Kaštel Gomilica, Cesta bb

DB, DV, Office of FM Split, Sučidar 79/II

MB, Office of FM Solin, Petra Krešimira IV 34

IP, Office of FM Split, M Getaldića 27

## Pre-publication history

The pre-publication history for this paper can be accessed here:

http://www.biomedcentral.com/1472-6920/13/114/prepub
